# An Approach to Biometric Verification Based on Human Body Communication in Wearable Devices

**DOI:** 10.3390/s17010125

**Published:** 2017-01-10

**Authors:** Jingzhen Li, Yuhang Liu, Zedong Nie, Wenjian Qin, Zengyao Pang, Lei Wang

**Affiliations:** Shenzhen Institutes of Advanced Technology, Chinese Academy of Science, Shenzhen 518055, China; lijz@siat.ac.cn (J.L.); yh.liu2@siat.ac.cn (Y.L.); wj.qin@siat.ac.cn (W.Q.); dl.yang@siat.ac.cn (Z.P.); wang.lei@siat.ac.cn (L.W.)

**Keywords:** biometric verification, human body communication, threshold-adaptive template matching, weighted Euclidean distance, transmission gain S21, wearable device

## Abstract

In this paper, an approach to biometric verification based on human body communication (HBC) is presented for wearable devices. For this purpose, the transmission gain S21 of volunteer’s forearm is measured by vector network analyzer (VNA). Specifically, in order to determine the chosen frequency for biometric verification, 1800 groups of data are acquired from 10 volunteers in the frequency range 0.3 MHz to 1500 MHz, and each group includes 1601 sample data. In addition, to achieve the rapid verification, 30 groups of data for each volunteer are acquired at the chosen frequency, and each group contains only 21 sample data. Furthermore, a threshold-adaptive template matching (TATM) algorithm based on weighted Euclidean distance is proposed for rapid verification in this work. The results indicate that the chosen frequency for biometric verification is from 650 MHz to 750 MHz. The false acceptance rate (FAR) and false rejection rate (FRR) based on TATM are approximately 5.79% and 6.74%, respectively. In contrast, the FAR and FRR were 4.17% and 37.5%, 3.37% and 33.33%, and 3.80% and 34.17% using K-nearest neighbor (KNN) classification, support vector machines (SVM), and naive Bayesian method (NBM) classification, respectively. In addition, the running time of TATM is 0.019 s, whereas the running times of KNN, SVM and NBM are 0.310 s, 0.0385 s, and 0.168 s, respectively. Therefore, TATM is suggested to be appropriate for rapid verification use in wearable devices.

## 1. Introduction

Body sensor networks (BSNs), which also referred to as body area networks (BANs), are wireless networks for interconnecting wearable nodes/devices centered on an individual person’s workspace [1,2]. With the rapid development of microprocessor technologies and wireless communication, BSNs have emerged as a revolutionary technology and have demonstrated great potential in healthcare monitoring (blood pressure monitoring [3], blood glucose monitoring [4], etc.), emotion recognition (negative emotional state of fear [5], etc.), sport performance monitoring [6], physical/virtual social interactions [7], and so on [8,9,10]. However, because wearable devices usually carry user’s personal information, information leakage from wearable devices in BSNs is regarded as a challenge, which may bring about an immeasurable loss [11]. Therefore, the information security of wearable devices should be strictly considered [12].

Biometric verification, which uses the human physiological or behavioral trait to achieve personal verification, is widely used in information security [13,14]. Compared with conventional verifications, such as digital password, personal identification number and IC card, biometric verification has the advantages of being much more difficult to forget, lose, steal, copy or forge [15]. Thus far, biometric verification using fingerprint, face, iris, vein, voice, electroencephalograph (EEG), electrocardiogram (ECG) and gait, among others, has been an active research topic in recent years [16,17,18,19]. Mathur et al. demonstrated the methodology of fingerprint verification in a wearable system [20]. However, the identification performance will be reduced when the finger is moist. Klonovs et al. introduced a mobile biometric verification system utilizing EEG recordings headset [21]. However, the EEG recordings headset is not suitable to wear for a long time. Peter et al. proposed an ECG-based authentication protocol to identify sensor nodes attached to the same human body [22]. Choudhary et al. presented a biometric verification approach based on the photoplethysmographic (PPG) signal for BSNs [23]. Derawi et al. collected the user’s gait as biometric trait through a wireless monitor [24]. However, the wireless monitor is so complicated that it is difficult to wear. Kim et al. presented a multimodal verification approach that uses face, teeth and voice modalities as biometric traits for mobile device [25]. However, the power of multimodal verification is too large to be used in wearable devices. Other biometric verifications, such as iris, hand and vein verification, are difficult to integrate into wearable devices due to the limitation of wearable devices’ size [26,27,28]. Therefore, a new approach to biometric verification is necessary in wearable devices [29].

Human body communication (HBC), which uses the human body itself as a transmission medium, provides a potential personal verification solution for wearable devices [30]. Specifically, due to the thickness differences of biological tissues in human body, the transmission gain S21, which reflects the variation of transmission characteristics at different frequencies, are different while the signal is coupled into the human body. Therefore, the transmission gain S21 may be used as a biometric trait to achieve personal verification. This is the theoretical foundation of biometric verification based on HBC. Considering that the location of a specified wearable device is usually fixed (e.g., a wristband is worn on the forearm), the HBC sensor, which is attached to the wearable device, can collect the biometric trait in the fixed location. In other words, the biometric verification based on HBC is readily integrated into different wearable devices. Thus, biometric verification based on HBC may be a promising technology in wearable devices [31].

Thus far, few investigations have characterized the biometric verification based on HBC. Nakanishi et al. presented a verification approach that uses a pseudo white noise as an input signal to acquire human biometric trait [32]. However, the identification performance is low due to the influence of randomness from white noise. Rasmussen et al. proposed a biometric based on the human body’s response to an electric square pulse signal, and used the pulse-response biometric as an additional verification mechanism [33]. However, all sample data are used in both learning and verification by the researchers, which may lead to a higher risk of confidence level. The authors of this article also made a preliminary research on biometric verification based on HBC [34]. However, the amount of computation in [34] is so large that it is inappropriate for rapid verification in wearable devices.

In this work, we aim to study the biometric verification based on HBC for wearable devices. The contribution and originality of this paper is summarized as follows. Firstly, the transmission gain S21 is proposed as the biometric trait for different individuals. Secondly, to achieve the rapid verification, a threshold-adaptive template matching (TATM) algorithm based on weighted Euclidean distance is employed. Furthermore, in order to evaluate TATM algorithm, the identification performance of TATM is compared with K-nearest neighbor (KNN) classification [35], support vector machines (SVM) [36], and naive Bayesian method (NBM) classification [37]. The remainder of this paper is organized as follows. In Section 2, we will demonstrate the validity of biometric verification method based on HBC through numerical simulation. In Section 3, the experimental setup will be introduced. Section 4 is about measurement result and analysis. TATM algorithm will be reported in Section 5. Section 6 gives a detailed analysis of identification performance. Finally, the conclusions are drawn in Section 7.

## 2. Modeling Biometric Verification Based on HBC

### 2.1. Forearm Modeling

**Figure 1:**
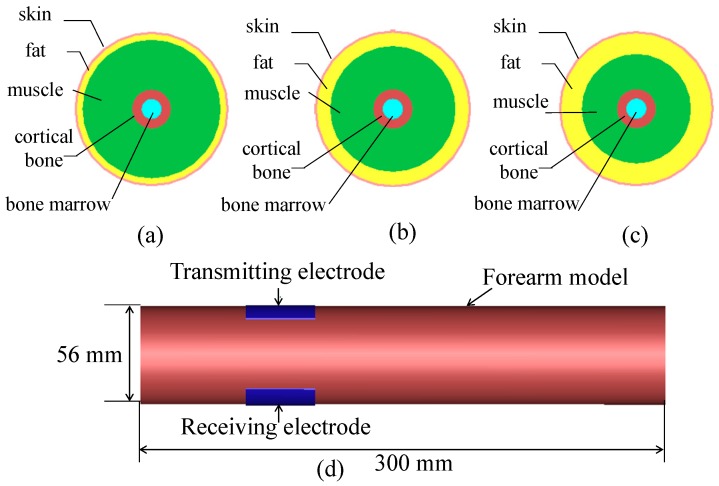
(**a**) The cross-section of Model A; (**b**) the cross-section of Model B; (**c**) the cross-section of Model C; and (**d**) transmitting electrode and receiving electrode.

As demonstrated in Figure 1, in order to evaluate the feasibility of biometric verification based on HBC, three different forearm models, namely, Model A, Model B and Model C, are established. These models are abstracted as cylinders. The length and diameter of all models are 300 mm and 56 mm, respectively. Furthermore, each model includes, from outside to inside, skin, fat, muscle, cortical bone and bone marrow [38]. The thicknesses of tissue layers for different models are listed in Table 1. Specifically, the thicknesses of fat and muscle for Model A are 2.30 mm and 17.86 mm, respectively. Compared with Model A, the thickness of fat in Model B is increased, whereas the thickness of muscle is decreased. In addition, the thickness of fat in Model C is 7.60 mm, and the thickness of muscle is about 12.56 mm. Details of simulation setup are as follows. A transmitting electrode and a receiving electrode are attached on the surface of model. A voltage source with an output impedance of 50 Ω is fed to the transmitting electrode. In order to acquire the transmission gain S21 in the frequency range 0.3 MHz to 1500 MHz conveniently, a Gaussian signal, of which the pulse width was 0.25 ns, is adopted in the simulation. In addition, there is a load with impedance of 50 Ω in receiving electrode. The simulations are performed using commercial electromagnetic modeling software XFDTD based on the finite-difference time-domain (FDTD) method.

### 2.2. Simulation Result

**Figure 2:**
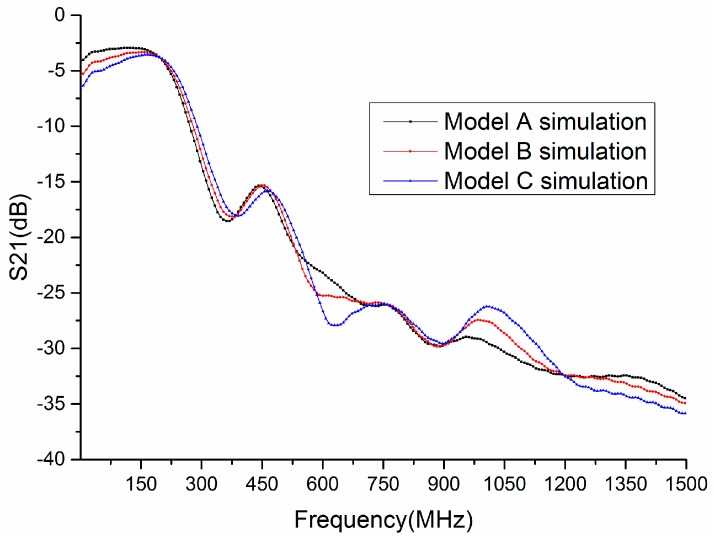
Transmission gain S21 of different models in FDTD simulations.

Figure 2 shows the transmission gain S21 (in dB) of three forearm models. The gains of three forearm models are somewhat different when the frequency is below 200 MHz. The gain of Model A is about −3 dB at 150 MHz, whereas the gain of Model C is −5.3 dB. The gains of all models are similar in the frequency range 200 MHz to 530 MHz. However, the gains are quite different when the frequency is 530 MHz to 750 MHz and 900 MHz to 1500 MHz. For instance, the gain of Model C is the smallest at 630 MHz, about −28 dB, whereas the gain of Model A is approximately −23 dB at 630 MHz. In addition, the gain of Model C is more than −26.5 dB at 1000 MHz, whereas the gain of Model A is about −30 dB. From Figure 2, it can be revealed that the transmission gain S21 of each forearm model is different, which is related with the thicknesses of tissue layers, as demonstrated in Figure 1. Thus, considering the difference of biological tissues for each individual, the transmission gain S21 is an optional biometric trait to achieve personal verification.

## 3. Experimental Setup

### 3.1. Experimental Equipment

**Figure 3:**
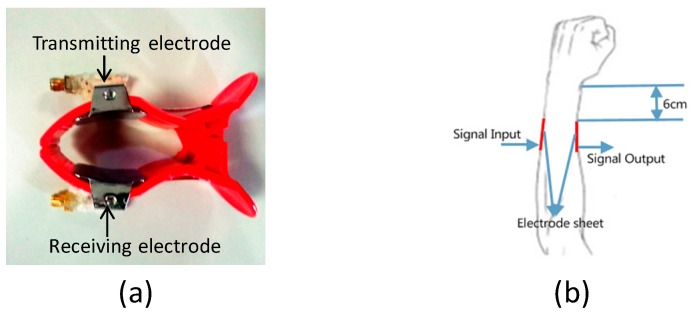
(**a**) Electrodes and plastic clip; and (**b**) measurement location.

The experimental equipment includes a vector network analyzer (VNA, Agilent E5061A), a transmitting electrode and a receiving electrode. In order to ensure that the electrodes are in close contact with the skin, the electrodes are attached on a plastic clip, as shown in Figure 3a. The VNA is adopted to acquire the transmission gain S21 of volunteer. Figure 3b illustrates the measurement location of volunteer. The transmitting electrode and receiving electrode are placed on the volunteer’s forearm. The distance between electrode and wrist is 6 cm. The transmitting electrode is connected to the Port 1 of VNA through cable. Similarly, the receiving electrode is connected to the Port 2 of VNA.

### 3.2. Experimental Setup

**Figure 4:**
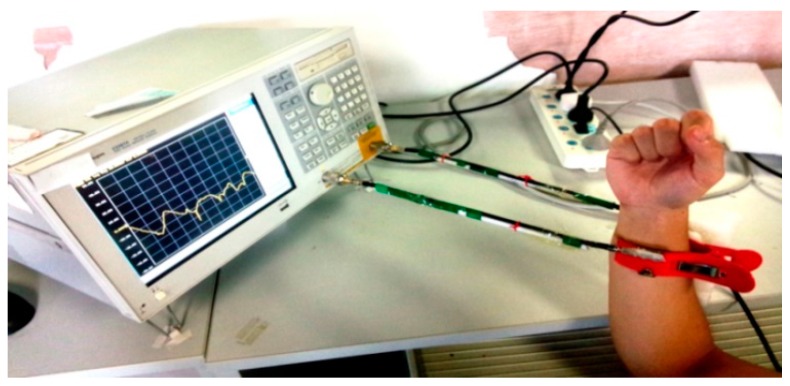
Experimental scenario.

In our study, ten volunteers (average age of 24 years) with body weights of 50 kg to 80 kg and body heights of 165 cm to 180 cm were selected. Written informed consent was obtained from all volunteers. Figure 4 shows the experimental scenario. Two experiments were carried out in this work.

Table 2 lists the detailed setup for Experiments 1 and 2. In Experiment 1, we aimed to find the chosen frequency for biometric verification based on HBC. For this purpose, the transmission gain S21 in the frequency range 0.3 MHz to 1500 MHz was investigated. The measurement was done 60 times (groups) per day for each volunteer and repeated for 3 days. Specifically, the measurement was done 10 times per hour for 6 hours each day. Moreover, 1601 sample data are acquired in each time. Thus, 2,881,800 sample data are acquired in Experiment 1.

According to Experiment 1, it can be known that the chosen frequency for biometric verification is from 650 MHz to 750 MHz. In Experiment 2, we aimed to obtain the sample data used for learning and verification at the chosen frequency. In Experiment 2, the measurement was done 6 times (groups) per day for each volunteer and was repeated for 5 days. Furthermore, 21 sample data are acquired each time. Therefore, 6300 sample data are acquired in Experiment 2.

## 4. Measurement Results and Analysis

### 4.1. Feasibility of Biometric Verification Based on HBC

**Figure 5:**
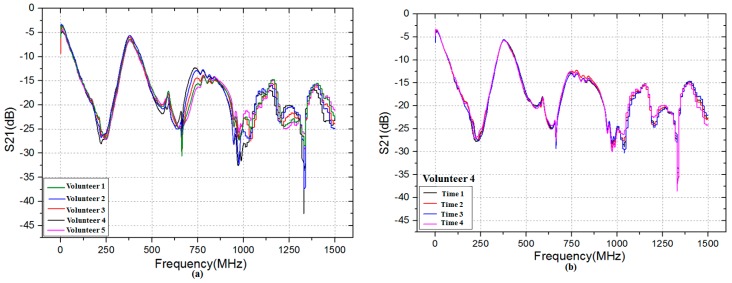
Transmission gain S21 in the frequency range 0.3 MHz to 1500 MHz: (**a**) five volunteers at the same time; and (**b**) Volunteer 4 at four different times.

Figure 5a depicts the transmission gain S21 (in dB) of five volunteers at the same time. As shown in Figure 5a, it is interesting to observe that there is a significantly difference among five volunteers in the frequency range 500 MHz to 1500 MHz. Moreover, as the frequency increases, the difference is become more discernible. Therefore, it can be inferred that the biometric verification based on HBC is feasible due to the difference of transmission gain S21 between individuals. Figure 5b describes the transmission gain S21 of one volunteer at four different times. It can be observed that the transmission gain S21 is almost the same when the frequency is from 0.3 MHz to 1000 MHz, which means that the transmission gain S21 of the same individual is steady over a period of time.

### 4.2. Chosen Frequency for Biometric Verification

In order to decrease the number of sample data, it is critical to determine the HBC frequency for biometric verification, which is of great benefit to achieve rapid verification in wearable devices. For this purpose, the standard deviation of transmission gain S21 for ten volunteers in the frequency range 0.3 MHz to 1500 MHz is investigated in this section. In addition, the standard deviation of transmission gain S21 for one volunteer at nine different times is also studied. The standard deviation calculation is shown as Equation (1).

(1) $$s_{i}={\left(\frac{\sum_{i=1}^{n}{\left(x_{i}-\bar{x}\right)}^{2}}{n-1}\right)}^{\frac{1}{2}}$$

where $s_{i}$ is the standard deviation, $x_{i}$ is the value of *i*-th sample data, $\bar{x}$ is the average value of sample data, and *n* is the sample times; *n* is 10 in the former calculation and *n* is 9 in the latter calculation.

**Figure 6:**
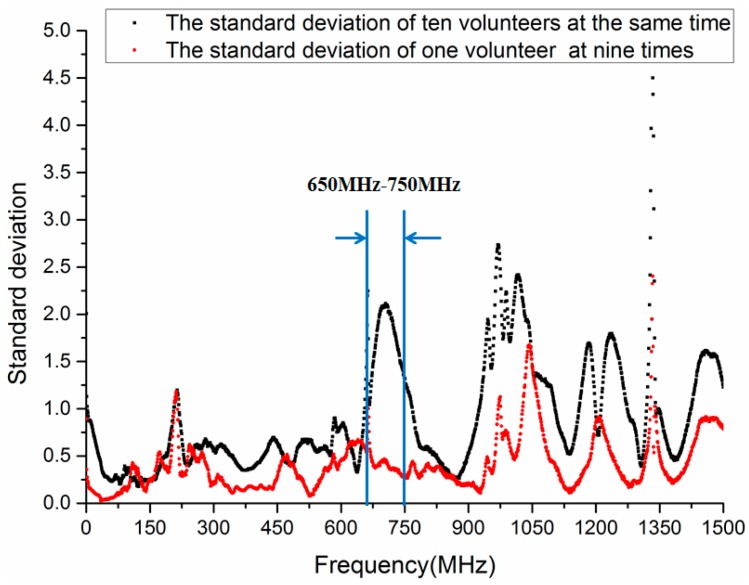
Standard deviation of transmission gain S21 in the frequency range 0.3 MHz to 1500 MHz.

Figure 6 illustrates the standard deviation of transmission gain S21 when the frequency is 0.3 MHz to 1500 MHz. The black curve represents the standard deviation among ten volunteers, and the red curve shows the standard deviation of a volunteer at nine different times. As demonstrated in Figure 6, the standard deviation among ten volunteers is equal to or less than 0.9 when the frequency is below 600 MHz. However, the standard deviation is greater than 1.3 when the frequency is 650 MHz to 750 MHz and 950 MHz to 1050 MHz. furthermore, the standard deviation is up to 2.1 at 700 MHz. Thus, it is indicated that there is a distinguishable difference among volunteers in aforementioned frequency range. On the other hand, the standard deviation of a volunteer (Volunteer 4) at nine different times is less than 0.6 in the frequency range 290 MHz to 950 MHz. As the frequency increases, the standard deviation is become larger. Thus, it can be deduced that the chosen frequency for biometric verification based on HBC should be from 650 MHz to 750 MHz, in which the standard deviation of ten volunteers is more than 1.3, but the standard deviation of a volunteer at nine different times is approximately 0.4.

In order to better understand the statistic characteristics of biometric verification based on HBC in the frequency range 650 MHz to 750 MHz, the coefficient of variation, which can reflect the relative dispersion of data, is adopted in this section. The calculation of coefficient of variation is shown as Equation (2).

(2) $$\mathrm{CV}=\left|\frac{s_{i}}{\bar{x}}\right|\times 100\%$$

where CV is the coefficient of variation, $s_{i}$ is the standard deviation of sample data, and $\bar{x}$ is the average value of sample data.

**Figure 7:**
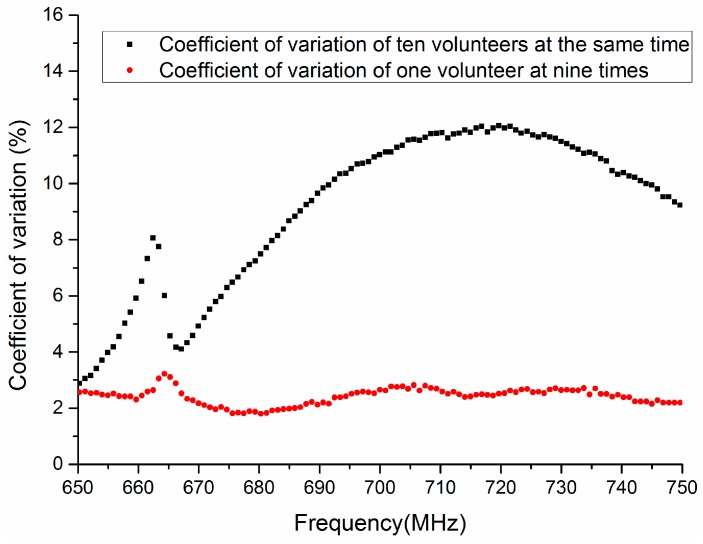
The coefficient of variation of transmission gain S21 in the frequency range 650 MHz to 750 MHz.

As shown in Figure 7, the coefficient of variation of ten volunteers is more than 8% when the frequency is 664 MHz. As the frequency increases, the coefficient of variation has a feature of sustained rise in the frequency range 665 MHz to 715 MHz. In addition, the coefficient of variation is 12.5% at 715 MHz and is 9.5% at 750 MHz. However, the coefficient of variation of one volunteer at nine different times is approximately 2.5% when the frequency is 650 MHz to 750 MHz. Thus, it is indicated that the frequency 650 MHz to 750 MHz may be appropriate for biometric verification based on HBC.

### 4.3. Transmission Gain S21 at Chosen Frequency

**Figure 8:**
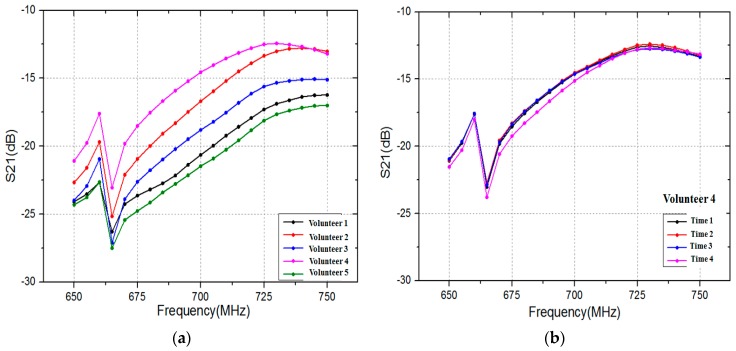
Transmission gain S21 in the frequency range 650 MHz to 750 MHz: (**a**) five volunteers at the same time; and (**b**) Volunteer 4 at four different times.

To achieve rapid verification, the number of sample data for human biometric trait should not be too high. In this paper, 21 sample data are acquired in each time (group) via VNA when frequency is from 650 MHz to 750 MHz. The frequency interval between each sample data is 5 MHz. Figure 8 shows the transmission gain S21 (21 sample data) in the frequency range 650 MHz to 750 MHz. As shown in Figure 8, there is a significantly difference among volunteers at the chosen frequency. In contrast, the difference of one volunteer at four times is small. Therefore, those 21 sample data can be regarded as human biometric trait.

## 5. TATM Algorithm Proposed

### 5.1. Template Building

**Figure 9:**
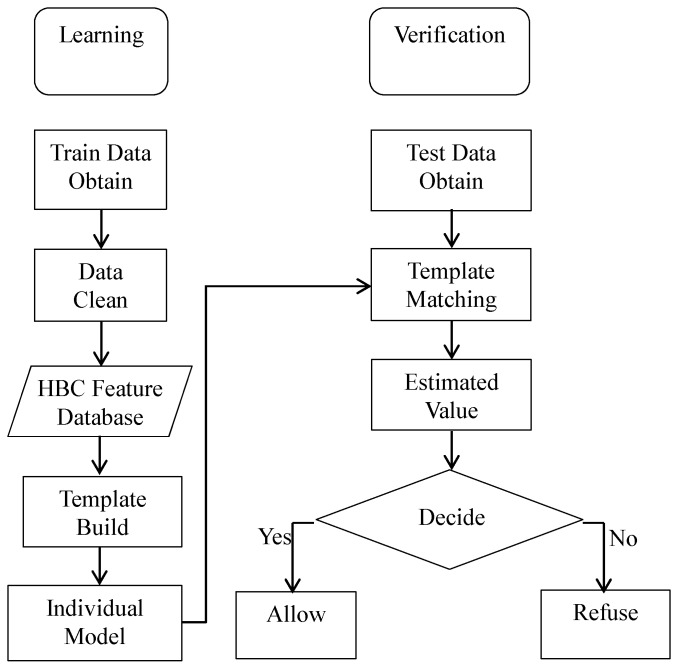
Flow diagram of TATM algorithm.

In this paper, a threshold adaptive template matching (TATM) algorithm based on weighted Euclidean distance is proposed to achieve personal verification. Figure 9 illustrates the process of the TATM algorithm. In general, the personal verification is divided into two steps: learning and verification [39]. In the first step, the error data need to be cleaned from the template library before the matching template is built. The second step is to determine the correlation between sample data and matching template. It is worth noting that the data used for matching template building and verification are obtained via Experiment 2. Specifically, 18 groups of data for each volunteer, obtained during the first three days, are used as template library to build the matching template, and the remaining groups (12 groups) are used to verification. Moreover, each group includes 21 sample data.

However, the sample data are unsteady in a certain range owing to the influence of VNA and ambient environment. Moreover, the change of experimental condition sometimes has a great impact on sample data. Therefore, the error data should be removed from the template library. In this paper, a simple and effective method is adopted to remove the error data from template library. Firstly, the sample data of 18 groups for each volunteer are taken as the initial template library *lib*1, as shown in Equation (3).

(3) $$\{\begin{array}{c} lib1={\left(\begin{array}{cccc} x_{11} & x_{12} & \cdots & x_{1n}\\ x_{21} & x_{22} & \cdots & x_{2n}\\ \vdots & \vdots & \ddots & \vdots \\ x_{m1} & x_{m2} & \cdots & x_{mn} \end{array}\right)}_{m\times n}\\ y_{j}=\frac{1}{m}\overset{m}{\underset{i=1}{\sum }}x_{ij},\;1\leq j\leq n\\ M_{1}={\left(\begin{array}{cccc} y_{1} & y_{2} & \cdots & y_{n} \end{array}\right)}_{1\times n} \end{array}$$

where m represents the number of feature vectors in each initial template library and the value of m is 18. n represents the number of feature points in each feature vector, and the value of n is 21. $M_{1}$ is the initial matching template which consists of 21 feature points.

Secondly, the Euclidean distance between the initial matching template $M_{1}$ and each feature vector that belongs to the template library is calculated. The feature vector will be excluded when the Euclidean distance is greater than the threshold $T_{1}$. The calculation of Euclidean distance is represented in Equation (4).

(4) $$\{\begin{array}{c} S_{i}={\left(\begin{array}{cccc} x_{i1} & x_{i2} & \cdots & x_{in} \end{array}\right)}_{1\times n}\\ S_{i}\in lib1,\;1\leq i\leq m\\ D_{1}={\left|⏐S_{i}-M_{1}⏐\right|}_{2}=\sqrt{{\left(S_{i}-M_{1}\right)}^{T}\left(S_{i}-M_{1}\right)}\\ D_{1}=\{\begin{array}{c} \geq T_{1},\;delete\\ \leq T_{1},\;save \end{array} \end{array}$$

where $S_{i}$ is the feature vector of initial template library lib1, $D_{1}$ is the Euclidean distance between $M_{1}$ and $S_{i}$, and $T_{1}$ is the Euclidean distance threshold. The value of threshold $T_{1}$ can be set to 2 in this paper.

A new template library *lib*2 is obtained after the error data are removed from *lib*1. Subsequently, the matching template $M_{2}$ is generated according to Equation (5). This template is the final feature vector of individual, which represents individual’s behavioral trait.

(5) $$\{\begin{array}{c} lib2={\left(\begin{array}{cccc} x_{11} & x_{12} & \cdots & x_{1n}\\ x_{21} & x_{22} & \cdots & x_{2n}\\ \vdots & \vdots & \ddots & \vdots \\ x_{\left(m-r\right)1} & x_{\left(m-r\right)2} & \cdots & x_{\left(m-r\right)n} \end{array}\right)}_{\left(m-r\right)\times n}\\ y_{j}^{\prime }=\frac{1}{m-r}\overset{m-r}{\underset{i=1}{\sum }}x_{ij}^{\prime },\;1\leq j\leq n\\ M_{2}={\left(\begin{array}{cccc} y_{1}^{\prime } & y_{2}^{\prime } & \cdots & y_{n}^{\prime } \end{array}\right)}_{1\times n} \end{array}$$

where *lib*2 is (m−r)×n Matrix, r is the number of feature vectors which have been cleaned, and $M_{2}$ is the matching template used for verification.

### 5.2. Verification

Considering that the weights of feature points are different in a feature vector, TATM based on weighted Euclidean distance is proposed in this work. Compared with classical Euclidean distance calculation method, the calculations are more precise. The calculation of TATM based on weighted Euclidean distance is performed as follows. The difference between maximum $max_{1i}$ and minimum $min_{1i}$ of sample data, which belong to template library *lib*2 at the same frequency, is calculated. The reciprocal of difference is used as the corresponding feature point weight $C_{i}$, as shown in Equation (6).

(6) $$\{\begin{array}{c} max_{1j}=\max\left(x_{1j}\;,\;x_{2j}\;,\;\cdots ,\;x_{\left(m-r\right)j}\right),\;0\leq j\leq n\\ min_{1j}=\min\left(x_{1j}\;,\;x_{2j}\;,\;\cdots ,\;x_{\left(m-r\right)j}\right),\;0\leq j\leq n\\ C_{j}=1/\left(max_{1j}-min_{1j}\right) \end{array}$$

According to the value of matching template $M_{2}$, the distance between the feature vector in template library *lib*2 and matching template $M_{2}$ is calculated. Then, the maximal value of distance $max_{2}$ is set to threshold $T_{2}$, as shown in Equation (7).

(7) $$\{\begin{array}{c} D_{2i}=\sqrt{\overset{n}{\underset{j=1}{\sum }}C_{j}{\left(x_{ij}-M_{2j}\right)}^{2}},\;0\leq i\leq m-r\\ max_{2}=\max\left(D_{21},D_{22},D_{23},\ldots ,D_{2\left(m-r\right)}\right)\\ T_{2}=max_{2} \end{array}$$

where *j* is the *j*-th feature point in a feature vector, and $D_{2i}$ is the value of Euclidean distance between matching template $M_{2}$ and *i*-th feature vector in *lib2*.

In terms of Equation (7), it is revealed that the threshold and weight are defined by matching template. The advantage of this method is that it does not required many experiments to find the suitable value. $T_{2}$ is utilized as the verification threshold in TATM algorithm to confirm whether the user is the authorized person. In verification mode, the test sample will be divided into two classes: the *I*-related and the *I*-non-related. Under the premise of the acceptable false acceptance rate (FAR), this method can get the smallest false rejection rate (FRR). The final determination condition is shown as Equation (8).

(8) $$\{\begin{array}{c} D=\sqrt{\overset{n}{\underset{j=1}{\sum }}C_{j}{\left(Fdata_{j}-M_{2j}\right)}^{2}}\\ D\{\begin{array}{c} \geq T_{2},\;I-\mathrm{non}-\mathrm{related}\\ <T_{2},\;I-\mathrm{related} \end{array} \end{array}$$

where $Fdata_{j}$ is the *j*-th sample data in test feature vector, and *D* is the distance between test data and matching template $M_{2}$.

## 6. Algorithm Evaluation

### 6.1. Effect of Data Cleaning

**Figure 10:**
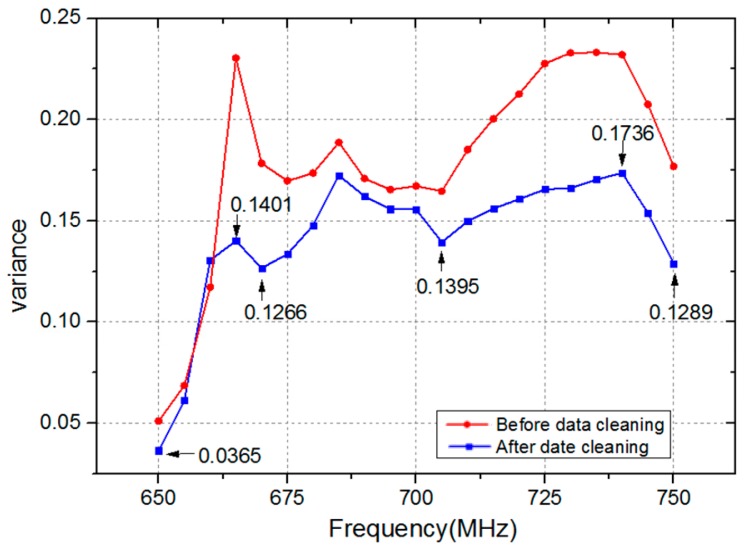
The variance of feature points after data cleaning.

Figure 10 demonstrates the variance of 21 feature points in the frequency range 650 MHz to 750 MHz. As demonstrated in Figure 10, the variance of feature points is reduced after data cleaning. The maximum and minimum values are 0.23 and 0.05 before data cleaning, and 0.1736 and 0.0365 after data cleaning, respectively. Therefore, the data cleaning method adopted in this paper is effective at removing error data from template library.

On the other hand, the variance of feature points is different at different frequencies after data cleaning. Specifically, the variances of these feature points are 0.0365, 0.1401, 0.1266, 0.1395, 0.1736, and 0.1289, respectively, at frequencies 650 MHz, 665 MHz, 670 MHz, 705 MHz, 740 MHz, and 750 MHz. It is revealed that feature points are not invariable values. A smaller variance of feature point means that the biometric trait is more stable, which is of great help to improve the accuracy of personal verification. Therefore, the weight of feature point of which the variance is relatively small should be set to a higher value to highlight its importance. In contrast, the weight should be decreased if the variance of feature point is large.

To better understand the influence of Euclidean distance threshold $T_{1}$ on data cleaning, the false acceptance rate (FAR) and false rejection rate (FRR) are acquired at different threshold $T_{1}$. FAR reflects the rate at which the imposters are accepted into the system, and FRR reflects the rate at which the authorized users are denied entry into the system. The calculations of FAR and FRR are shown as Equations (9) and (10). Table 3 lists the FAR and FRR at different Euclidean distance threshold $T_{1}$.

(9) $$\mathrm{FAR}=\frac{false\;acceptance\;samples}{total\;acceptance\;samples}\times 100\%$$

(10) $$\mathrm{FRR}=\frac{false\;rejection\;samples}{total\;acceptance\;samples}\times 100\%$$

As listed in Table 3, the Euclidean distance threshold $T_{1}$ has a great impact on data cleaning when it is less than 5. Furthermore, the FAR is 5.79% and the FRR is 6.74% when the Euclidean distance threshold $T_{1}$ is equal to 2. However, the FRR is up to 36.8% and 13.3%, respectively, when the Euclidean distance threshold $T_{1}$ is 1.5 and 2.5. Therefore, considering the relatively low FAR and FRR, the Euclidean distance threshold $T_{1}$ can be set to 2 in this paper.

### 6.2. The EER

**Figure 11:**
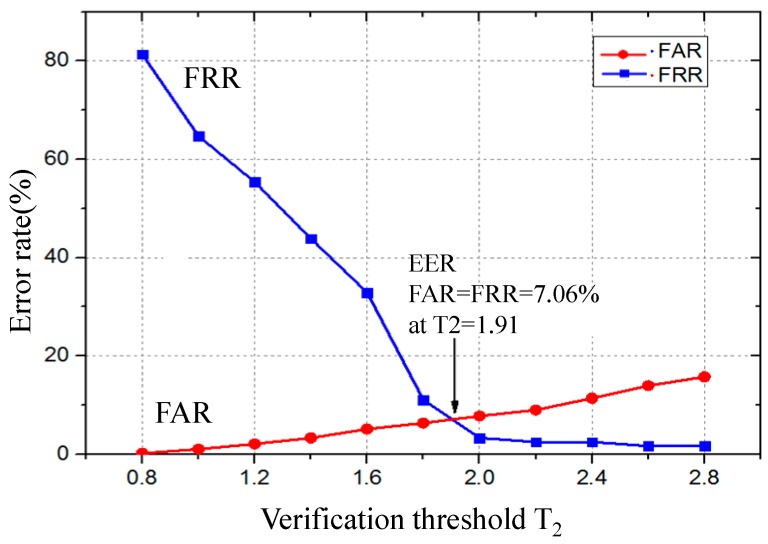
FAR and FRR for different verification threshold $T_{2}$.

In this section, 120 groups of data obtained during Days 4 and 5 in Experiment 2 are adopted to calculate the FAR, FRR and equal error rate (EER). Figure 11 shows the values of FAR and FRR when the verification threshold $T_{2}$ is from 0.8 to 2.8. As shown in Figure 11, the range of FRR is from 80% to 0.5%, and the range of FAR is from 0.1% to 18%. Furthermore, the EER is 7.06% when the FAR is equal to FRR at verification threshold $T_{2}=1.91$.

### 6.3. Algorithm Comparison

In this paper, the TATM based on weighted Euclidean distance is compared with K-nearest neighbor (KNN) classification, support vector machines (SVM) and naive Bayesian method (NBM). All algorithms are implemented by MATLAB on a personal computer. Furthermore, the SVM is achieved by the LIBSVM which is a library designed by Taiwan University [40], and the KNN and NBM are acquired from MATLAB function library. A total of 120 groups of sample data are used as the test data. The FAR and FRR of different algorithms are listed in Table 4. Additionally, the running time of algorithm is listed in Table 5.

As illustrated in Table 4, for Volunteers 1, 4, and 10, KNN shows a good performance of low FAR and FRR. However, KNN has a disappointing result for other volunteers. The FRR is more than 50% for Volunteers 3, 6, 7, and 9. Thus, the KNN is unsuitable for biometric verification based on HBC. The FRR obtained by SVM is more than 10% for Volunteers 2, 3, 5, 6, 7, 8, and 9. Moreover, the FRR of Volunteers 7 and 9 is 91.67%. The FRR of SVM is so large that it is inappropriate for verification. Similarly, the NBM also shows a bad performance, and the FRR is greater than 10% for eight volunteers in the measurement. On the other hand, the TATM shows a good performance for different volunteers (Table 4). The average values of FAR and FRR are 5.79% and 6.74%, respectively. Furthermore, as listed in Table 5, the running time of TATM is the shortest (0.019 s), whereas the running time of KNN is up to 0.310 s, while the running times of SVM and NBM are 0.0385 s and 0.168 s, respectively. Thus, it can be concluded that the TATM is more suitable for rapid verification owing to it has lower FRR and shorter running time.

**Figure 12:**
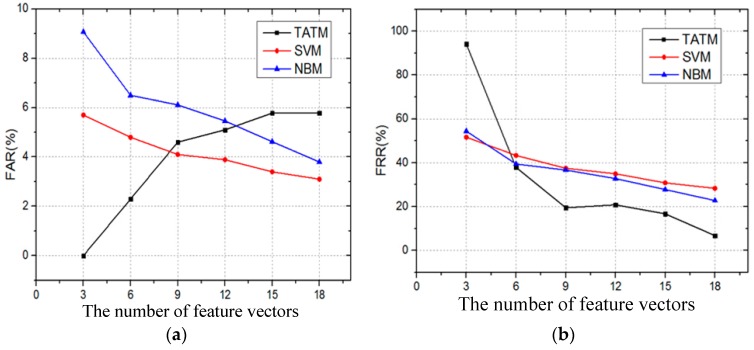
The influence of the number of feature vectors: (**a**) FAR; and (**b**) FRR.

The influence of the number of feature vectors on FAR and FRR is investigated next. Figure 12 describes the FAR and FRR with different numbers of feature vectors. In Figure 12a, it can be observed that the FAR of three algorithms is less than 6% when the number of feature vectors is 18. Additionally, as demonstrated in Figure 12b, the FRR is sensitive to the numbers of feature vectors. The FRRs of all algorithms are greater than 50% when the numbers of feature vectors is equal to 3. However, the FRR of SVM and NBM is approximately 25%, and the FRR of TATM is only 6.74% when the number of feature vectors is 18. Thus, compared with SVM and NBM, TATM presents a lower FRR.

Table 6 lists the EER, running time, and computational memory of biometric verification based on HBC in previous works. In [34], the EER of 0.56% is achieved by SVM. However, the number of feature vectors is 160, and the number of feature points for each feature vector is up to 1600. Furthermore, the running time and computational memory of SVM in Reference [34] are approximately 9.941 s and 91 MB. In Reference [31], the EER of 25% is obtained by SVM when 40 feature vectors and each feature vector includes 100 feature points are adopted. In our paper, the EER of 7.06% is achieved by TATM when 18 feature vectors are utilized. Furthermore, the number of feature points in each feature vector is only 21. Additionally, the running time and computational memory of TATM in this article are 0.019 s and 2 MB, respectively. Thus, it is concluded that TATM can provide a rapid verification with a relatively low running time and computational memory.

### 6.4. Discussion

As listed in Table 4, it is worth noting that both the FAR and FRR of Volunteer 10 are equal to zero, which may be associated with the volunteer herself. Specifically, the forearm of Volunteer 10 is thinner than the other volunteers, which led to her transmission gain S21 being quite different.

On the whole, as listed in Table 4, The FRR of KNN, SVM, and NBM is high even when the parameters of algorithms were varied, which may be related with the number of training data [27]. In the learning groups, the training data for each volunteer (18 groups of data) are fewer than those of others (162 groups of data), so a volunteer’s classification area is narrower and might overlap with those of other volunteers, which leads to a high FRR.

However, the FRR of TATM is relatively low, which is associated with the verification threshold $T_{2}$. The verification threshold $T_{2}$ is threshold-adaptive in this paper, namely, the verification threshold $T_{2}$ of each volunteer is mainly dependent on the number of each volunteer’s own training data rather than those of others. Thus, the TATM shows low FRR.

## 7. Conclusions

This paper proposes a rapid biometric verification for application in wearable devices. The transmission gain S21 of individual is measured in the frequency range 0.3 MHz to 1500 MHz. The results indicate that there is significantly different transmission gain S21 among individuals, and the transmission gain S21 for the same individual is steady over a period of time. Furthermore, it is also revealed that the chosen frequency for biometric verification based on HBC is 650 MHz to 750 MHz. In addition, a threshold-adaptive template matching (TATM) algorithm based on weighted Euclidean distance is proposed in this paper. In order to achieve rapid verification, 18 groups of data, each group including 21 sample data, are used as the template library. In terms of template library, the matching template used for personal verification is built after the data cleaning. Meanwhile, the weights of feature points are calculated. The results show that the TATM algorithm presents a good performance with relative lower FAR and FRR, 5.79% and 6.74%, respectively. In contrast, the FAR and FRR were 4.17% and 37.5%, 3.37% and 33.33%, and 3.80% and 34.17%, respectively, using KNN, SVM, and NBM. In addition, the running time of TATM is the shortest (0.019 s), while the running times of KNN, SVM and NBM are 0.310 s, 0.0385 s, and 0.168 s, respectively. Compared with other algorithms, the TATM based on weighted Euclidean distance has lower FRR and shorter running time. Therefore, the TATM proposed in this paper may be a potential solution for rapid verification for wearable devices. In the near future, the biometric verification based on HBC and the TATM algorithm will be achieved in a wearable prototype.

## Figures and Tables

**Table 1:** Thicknesses of difference tissue layers (mm).

	Model A	Model B	Model C
Skin	0.84	0.84	0.84
Fat	2.30	4.76	7.60
Muscle	17.86	15.4	12.56
Cortical bone	3.36	3.36	3.36
Bone marrow	3.64	3.64	3.64

**Table 2:** Experimental setup.

	Frequency Bands	Volunteers	Days	Times per Day	Sample Data per Time	Total
Experiment 1	0.3 MHz–1500 MHz	10	3	60	1601	2,881,800
Experiment 2	650 MHz–750 MHz	10	5	6	21	6300

**Table 3:** Influence of Euclidean distance threshold $T_{1}$.

Threshold $T_{1}$	1	1.5	2	2.5	3	3.5	4	5	6	7
FAR	1.09%	5.24%	5.79%	8.26%	15.2%	14.5%	17.6%	17.4%	17.4%	17.4%
FRR	77.7%	36.8%	6.74%	13.3%	3.33%	4.17%	4.17%	5.0%	5.0%	5.0%

**Table 4:** FAR and FRR of different algorithms.

Volunteer	TATM		KNN		SVM		NBM	
	FAR	FRR	FAR	FRR	FAR	FRR	FAR	FRR
1	0.95%	0	7.41%	8.33%	0	0	0	16.67%
2	15.24%	0	8.33%	33.33%	7.41%	58.33%	7.41%	41.67%
3	3.81%	25%	4.63%	66.67%	5.56%	25.0%	10.19%	41.67%
4	1.89%	0	3.70%	8.33%	2.78%	8.33%	3.70%	8.33%
5	0	8.33%	0	25%	0	33.33%	0	41.67%
6	13.21%	0	6.48%	50.0%	5.56%	8.33%	4.63%	25.0%
7	6.67%	25%	0.93%	58.33%	1.85%	91.67%	0.93%	25.0%
8	3.77%	9.09%	3.70%	33.33%	10.19%	16.67%	3.70%	41.67%
9	12.38%	0%	6.48%	91.67%	3.70%	91.67%	7.41%	100%
10	0	0	0	0	0	0	0	0
Average	5.79%	6.74%	4.17%	37.5%	3.37%	33.33%	3.80%	34.17%

**Table 5:** Running time of different algorithms

Algorithm	TATM	KNN	SVM	NBM
Running time (s)	0.019	0.310	0.0385	0.168

**Table 6:** Comparison with previous works.

	[34]	[31]	This Article
The number of feature vectors	160	40	18
Feature point in each feature vector	1600	100	21
Algorithm	SVM	SVM	TATM
EER	0.56%	25%	7.06%
Running time	9.941 s	-	0.019 s
Computational memory	91 MB	-	2 MB

## Appendix A

The algorithms of KNN, NBM and SVM are introduced as follows.

### A.1. KNN

KNN is usually used for clustering with the advantage of simple principle and fast running speed for large datasets. The main idea of KNN is that the datasets are divided into different categories by the iterative process. The detailed steps for KNN are as follows.

(1) The dataset X and the number of clustering N (N > 1) are set at initialization time. N objects are randomly selected as the initial cluster centers in dataset X.
(2) The Euclidean distance between each object and the cluster centers is calculated. According to the principle of minimum distance, datasets will be divided into N classes again.
(3) The average value of each class is used as a new clustering center.
(4) If the new clustering center is equal to the cluster center, the iterative process stops; otherwise, repeat Step 2 and Step 3.

### A.2. NBM

NBM, which is based on the probability density function, is an efficient and simple classification algorithm. NBM can be used to describe the relation between conditional probability and classification in the system, as shown in Equation (A1).

(A1) $$P\left(B|A\right)=\frac{P\left(A|B\right)P\left(B\right)}{P\left(A\right)}$$

Compared with the other classification methods, NBM has lower time complexity and higher accuracy. As a well-developed classification method, NBM has been widely used in classification. The steps of NBM are as follows.

(1) $X=\left\{a_{1},a_{2},\ldots ,a_{m}\right\}$ and $C=\left\{y_{1},y_{2},\ldots ,y_{n}\right\}$ are set as a sorting item and collection of categories, respectively. C is trained in advance.
(2) The Conditional probability $P(y_{i}|X)$ for the sorting item X is calculated by Equation (A2).
  (A2) $$P\left(y_{i}|X\right)=\frac{P(X|y_{i})}{P\left(X\right)},\;0<i\leq n$$
(3) The value of $P\left(y_{k}|X\right)$ is obtained through Equation (A3).
  (A3) $$P\left(y_{k}|X\right)=\max\left\{P\left(y_{1}|X\right),P\left(y_{2}|X\right),\ldots P\left(y_{n}|X\right)\right\}$$

### A.3. SVM

SVM is a data mining algorithm based on statistical theory. The mechanism of SVM is to obtain an optimal separating hyperplane to meet the requirements of classification. The optimal separating hyperplane ensures the accuracy of the classification. In addition, it has the largest distance of the classification point at optimal separating hyperplane. The two-class classification problem is taken as an example.

First, a training data set is acquired, as shown in Equation (A4).

(A4) $$\{\begin{array}{c} \left(x_{i}\;,\;y_{i}\right),\;i=1,2,\ldots ,l\\ x\in R,\;y_{i}=\pm 1 \end{array}$$

To solve two-class classification problem, the separating hyperplane is obtained.

(A5) ω⋅x+b=0

In order to classify all the samples correctly, the Equation (A6) needs to be satisfied.

(A6) $$y_{i}\left[\left(\omega \cdot x_{i}+b\right)\right]\geq 1,\;i=1,2,\ldots l$$

The classification interval is set to 2/||ω||. Thus, the problem of constructing the hyperplane is transformed into solving the constraint problem.

(A7) $$\min\emptyset \left(x\right)=\frac{1}{2}{\left|\left|\omega \right|\right|}^{2}=\frac{1}{2}\left(\omega^{\prime }\cdot \omega \right)$$

In order to solve the constrained optimization problem, the Lagrange function is introduced, as shown in Equation (A8).

(A8) $$L\left(\omega ,b,a\right)=\frac{1}{2}\left|⏐\omega ⏐\right|-a\left(y\left(\left(\omega \cdot x\right)+b\right)-1\right)$$

where a>0 is Lagrange multiplier. The solution of the optimization problem is determined by the saddle point of the Lagrange function. This solution’s partial derivatives of *b* and ω are 0 at the saddle point. The quadratic programming (QP) problem is transformed into a dual problem.

(A9) $$\{\begin{array}{c} \mathrm{maxQ}\left(a\right)=\overset{l}{\underset{j=1}{\sum }}a_{j}-\frac{1}{2}\overset{l}{\underset{i=1}{\sum }}\overset{l}{\underset{j=1}{\sum }}a_{i}a_{j}y_{i}y_{j}\left(x_{i}\cdot x_{j}\right)\\ s.t.\;\overset{l}{\underset{j=1}{\sum }}a_{j}y_{j}=0\;i,j=1,2,\ldots ,l\;a_{j}\geq 0 \end{array}$$

The solution is obtained by Equations (A8) and (A9).

(A10) $$\{\begin{array}{c} a^{*}={\left(a_{1}^{*},a_{2}^{*},\ldots ,a_{l}^{*}\right)}^{T}\\ \omega^{*}=\overset{l}{\underset{j=1}{\sum }}a_{j}^{*}y_{j}x_{j}\\ b^{*}=y_{i}-\overset{l}{\underset{j=1}{\sum }}y_{j}a_{j}^{*}\left(x_{j}\cdot x_{i}\right). \end{array}$$

Finally, the optimal classification function is obtained according to Equation (A5) and (A10).

(A11) $$f\left(x\right)=\mathrm{sgn}\left\{\left(w^{*}\cdot x\right)+b^{*}\right\}=\mathrm{sgn}\left\{\left(\overset{l}{\underset{j=1}{\sum }}a_{j}^{*}y_{j}\left(x_{j}\cdot x_{i}\right)\right)+b^{*}\right\}$$
